# Analysis of Psychological Risks in the Professional Activities of Oil and Gas Workers in the Far North of the Russian Federation

**DOI:** 10.3390/bs8090084

**Published:** 2018-09-19

**Authors:** Yana Korneeva, Natalia Simonova

**Affiliations:** 1 Department of Psychology, Northern (Arctic) Federal University named after M.V. Lomonosov, Arkhangelsk 163002, Russia; 2 Laboratory of Labor Psychology of the Faculty of Psychology, Moscow State University named after M.V. Lomonosov, Moscow 125009, Russia; n23117@mail.ru

**Keywords:** psychological risk, shift work, oil and gas production, functional status, character accentuation, adaptation

## Abstract

The professional activity in shifts in the Arctic contributes to the development of unfavorable functional status and destructive personal qualities of workers, which leads to a decrease in the level of mental health and efficiency of labor activity. The reference to the risk-oriented approach is conditioned by the need to predict the professional efficiency of shift personnel. The purpose of this study is to determine the psychological risks of oil and gas workers with a shift work organization in the Arctic. The study involved 70 oil and gas workers. The research methods were used as follows: documentation study, work process monitoring, questionnaire survey, psychophysiological and psychological testing, and statistical analysis methods: descriptive statistics—conjugacy tables with calculation of Pearson’s criterion, two-stage cluster, dispersion, and discriminant analyzes. As a result of this research, it was established that oil and gas workers characterized by different combinations of character accentuations would have different psychological risks, and, consequently, different approaches to their psychological support are needed.

## 1. Introduction

The development of mineral deposits in the Far North and the Arctic is a priority policy direction of the Russian Federation [1].

The regions of the Far North and the Arctic are characterized by extreme natural and climatic conditions and living conditions (group isolation, remoteness from the main industrial centers, high resource intensity of economic activity and life support of the population, high labor intensity), therefore, the shift work method is used in these territories. The shift method is a special form of implementation of the labor process outside the place of permanent residence of workers, when their daily return to their place of permanent residence cannot be provided [2].

Climatic–geographical, industrial, and social factors of the environment make physical and mental demands on shift workers that exceed their reserves and reduces the potential for workers to adapt to these extreme conditions and causes the presence of occupational risks. The risk is a likelihood of an adverse reaction, but this is only a probability that can become a reality if the level of negative impact exceeds the adaptive capabilities of the person and the capabilities of the defenses of the labor post.

The workers are also affected by a number of social and domestic factors: group isolation; forced character of interaction not only during production activities, but also on vacation; long separation from relatives; restriction in freedom of choice of the circle of contacts; restriction in freedom of movement; integration of the formal and informal structure of the shift team due to the tightness of contacts, relative isolation from other groups; publicity (impossibility of seclusion); information exhaustion; coordination of actions during the performance of production assignments; compatibility in communication during off-work time; and narrowing of the personal field. All these factors contribute to the development of special psychological risks in the professional work of shift workers. For the purpose of forecasting the efficiency of shift workers’ activities, it is necessary to determine the probability of occurrence of negative psychological conditions, qualities, and personality qualities that will impede its implementation. This goal can be achieved using a risk-based approach.

Psychological risk in professional activity is a probability of the emergence of professional personal destruction and the formation of unfavorable functional states of employees in the performance of labor functions due to the prolonged impact of negative social and industrial factors with insufficient personal and environmental resources [3,4].

Abroad, problems of workers in extractive industries are studied, mainly in Canada, Australia, Norway, Austria, the United States, France, etc. One of the key issues is the sleep disturbance of shift workers [5,6,7,8]. The works emphasize that shift work has many physiological, psychological, and social consequences that lead to disturbances in the normal sleep–wake cycle.

Generalization of the results of psychological research on the impact of negative factors of shift work on the psychological state and personality of specialists [9,10,11,12,13,14], made it possible to identify the unfavorable functional status and character accentuation as criteria for the psychological risks of shift workers in the Arctic. Taking into account the functional connections of the oil and gas workers’ functional status with the whole spectrum of personal characteristics, and also the identification of the functional status and character accentuations as the main risk criteria, we found it necessary to evaluate their interrelationships outside the context of other personal characteristics.

The purpose of the study is to determine the psychological risks of oil and gas workers with the watch-based organization of work in the Arctic.

The research hypotheses:

**Hypothesis 1** **(H1).** We expect a difference in character accentuations for shift workers with different levels of functional status (optimal and unfavorable).

**Hypothesis 2** **(H2).** The qualitative variety of combinations of different manifestations of character accentuations can be divided into statistically reliably different clusters (groups).

**Hypothesis 3** **(H3).** These groups will differ statistically and reliably in the levels of functional status.

## 2. Materials and Methods

To achieve this goal, a study was carried out at the oil and gas production facility with watch-based organization of work in the territory of the Nenets Autonomous District of Russia. The shift period lasts 30 days and 30 days of rest; 12-h day; there are no days off during the shift period. The study involved 70 workers aged 24 to 60 years (average age 38.7 ± 1.3). The work experience of shift workers under study varies from 0.5 to 31 years (9.53 ± 1.2).

The criteria for the formation of the sample were operators for oil and gas production, boiler operators, drivers, engineers, and maintenance workers. In the distribution of the subjects in groups, there was no randomization. The criteria for inclusion in the sample was the written informed consent of the patient to participate in the study, specially developed for this purpose. The criteria for non-inclusion were refusal to participate in the study and night shift. The type of research is analytical.

The research methods include studying documentation, monitoring the work process, questionnaire survey, psychophysiological and psychological testing, and statistical methods of data analysis. The developed questionnaire was aimed at obtaining information about the employees’ biographical details and special aspects of their work activity. It included the following sections:

**–**  general information on education and work experience;
**–**  marital status;
**–**  subjective assessment of the unfavorability of climatic, geographical, industrial, and social factors that affect workers during the rotation camp;
**–**  features of organizing leisure time during the shift period;
**–**  subjective assessment of their professional effectiveness and professionalism;
**–**  subjective assessment of the dangers of various situations that may occur during the shift period;
**–**  subjective hazard assessment in the workplace and factors contributing to its formation.

All methods and methods of the study, as well as the final article, were considered at the expert council of the Higher School of Psychology, Pedagogy and Physical Culture of the NArFU for observing the ethics of scientific research and the possibility of an open publication of these results.

To achieve the objectives of the study, the following methods were used:

1. Complex visual-motor reaction (SZRM), performed by means of the psychophysiological testing device UPFT-1/30 ‘Psychophysiologist’, which allows us to assess the level of mental and operational performance, as well as the level of functional status.

2. Variational cardiointervalometry (VKM), performed by means of the psychophysiological testing device UPFT-1/30 ‘Psychophysiologist’. This is an assessment of the functional status and adaptive capabilities of the cardiovascular system using the method of variational cardiointervalometry. These indicators are a vulnerable link in the adaptation to the conditions of the Far North and the Arctic with polar stress syndrome [15,16].

Evaluation of the functional status using these instrumentation techniques was carried out three times: in the beginning—1–3 days; in the middle—15–17 days; and at the end of 25–27 days of the shift period.

3. The method of “accentuation of character” G. Shmishek, K. Leonhard [17,18,19,20].

Statistical methods of analysis: descriptive statistics; multidimensional variance analysis, two-stage cluster analysis, and conjugacy tables using the X2-Pearson criteria. 

Statistical processing of data was carried out using the statistical package IBM SPSS Statistics 22.00 (license agreement no. Z125-3301-14 (Northern (Arctic) Federal University)).

## 3. Results

According to the measurements of the ‘Psychophysiologist’ device using the methods of ‘complex visual-motor reaction’ and ‘variational cardiointervalometry’, one of the levels of the functional status is determined in the subjects: (1) negative, (2) critical, (3) maximum permissible, (4) permissible, (5) close to optimal, and (6) optimal. The distribution of the workers by these groups was such that it did not allow to assess statistical differences, therefore we divided all the surveyed workers into two groups: those with an unfavorable functional status (those employees who had a negative, critical, or maximum permissible status) and with an optimal functional status (those employees who had a permissible, close to optimal, and optimal status).

As can be seen from the data in Table 1, by the middle of the shift period, there is an increase in the number of employees with an optimal level of functional status, which may be due to the end of the period of workability (i.e., the initial stage of the dynamics of working capacity, during which the person enters into work) and the onset of a period of optimum performance.

According to the descriptive statistics (Table 1), in the sample of workers surveyed, there are those who manage to keep their functional state optimal, and those who completely spend their internal resources and do not have time to fill them up during the between shifts holidays. It is this second group that makes up the risk group. A reduced level of the functional state may in the future lead not only to a decrease in efficiency and productivity, but also to a deterioration in the state of health.

We assumed that such a division into groups could be due to the presence of pronounced character accentuations. To test our proposal, we conducted a step-by-step discriminant analysis, where as the dependent variable it was the attribution to the group by the level of the functional status, and the independent one—the severity of the types of character accentuations. A stepwise option of discriminative analysis was used, so in the final version there remained variables that reached significant values of λ-Wilks (λ = 0.839 at *p* = 0.021). According to the values of the canonical function in group centroids, the maximum distinctive ability belongs to the single variable—the intensity of the demonstrative type of accentuation. The step-by-step method excluded from the analysis the following variables: hyperthymic, stuck, pedantic, emotive, anxious, cyclothymic, excitable, dysthymic, and exalted types.

The coefficient of the standardized canonical discriminant function is 1.0. The value of the function in the centroids of the group with an unfavorable functional state is 0.494, for the group with the optimal functional state it is minus 0.364. Data from descriptive statistics make it possible to conclude that workers with an unfavorable functional condition are characterized by an expression of more than 14 points of demonstrative character accentuation, while among employees with an optimal functional state this accentuation is expressed within the norm. The presence of demonstrativeness as a personal accentuation is traditionally characterized by emotional liveliness, artistry, and mobility; the desire to be in sight; a thirst for attention to oneself. In shift working conditions, it is very difficult to satisfy these needs (since work assumes mainly individual work, and a relatively small number of people work and reside in the oil industry), among others, people with this accentuation of personality traits like alterations and changes in everything, which is also impossible amid uniform and monotonous work in group isolation conditions. The lack of opportunity to show activity and draw attention to oneself in conditions of prolonged group isolation and depletion of resources, as well as unwillingness to bear responsibility for negative events at work and moving away from them to diseased state can lead to a decrease in the body’s functional reserves and the appearance of unfavorable conditions.

According to the data of descriptive statistics (Table 2), workers manifest most types of character accentuations.

We assumed that the sample of workers can be clustered according to a specific combination of the expression of character accentuation peculiar to oil and gas production workers in the Arctic. To test this assumption, we used a two-stage cluster analysis of 10 variable-types of character accentuations. As a result of the analysis, two clusters, corresponding to groups of specialists, statistically significantly different in the totality of types of character accentuations, were identified. For the most complete description of the differences in clusters, a multidimensional analysis of variance was applied, where groups of workers were assigned based on the results of cluster analysis and the severity of types of character accentuations. The results are shown in Table 3 and Table 4.

According to the multidimensional tests (Pillai trace = 0.843 for *p* < 0.001; Lambda Wilks = 0.157 for *p* < 0.001), there are statistically significant differences in the influence of the assignment of workers by the severity of character accentuations and types of character accentuations (*p* < 0.05).

According to the data of one-dimensional tests (Table 3), the differences between clusters are observed in the following parameters: stuck, emotive, anxious, cyclothymic, dysthymic, demonstrative, and exalted types (*p* < 0.05).

As can be seen from the data in Table 4, the employees of the first cluster are characterized by a higher severity of stuck, cyclothymic, demonstrative types, and a high level of exalted type of character accentuation. This is characterized by high efficiency, high level of claims, persistence in achieving the goal and the ability to defend their point of view. At the same time, such employees are capable of empathy, sociable, emotional, good-natured, and inspire confidence.

At the same time, such people are characterized by resentment, arrogance, lust for power, suspicion and rancor, in some situations may exhibit adventurism (a tendency to unjustified risk). Intricacies can be caused by long and careful work. The results of the work depend on the mood swings. With a bad mood, outbursts of irritability, and temper are possible.

Stressogenic for such people are situations of uncertainty, rigidly regulated conditions of activity, monotonous work, as well as underestimation of merits and indifference. Adverse conditions are also caused by a lack of communication and separation from loved ones.

Thus, the workers of the first cluster can be called as having an exalted demonstratively-stuck style of character accentuations.

Employees of the second cluster are distinguished by the presence of pronounced anxious, cyclothymic and dysthymic types of character accentuations. This manifests itself in a high degree of self-criticism, responsibility, commitment; strict adherence to norms, rules, independence in judgments, the existence of one’s own position.

At the same time, these workers are vulnerable, indecisive, cautious, pessimistic, and passive. They are characterized by the low stability in stressful situations, a feeling of helplessness, uncertainty, powerlessness in the face of external factors, as well as inertness and slowness of reactions, which makes it difficult to work under changing conditions.

Stress-related situations for such employees: an unfamiliar circle of contacts; the need to make decisions in conditions of the lack of information, a time limit; situations requiring mobilizing efforts, endurance and self-confidence (public speech, exams, competitions, etc.), as well as unfair accusations and mockery.

Thus, the employees of the second cluster can be called as having an anxious-cyclothymic style of character accentuations.

## 4. Discussion

The revealed peculiarities of the combination of accentuations of the character of employees of both clusters allow us to analyze possible psychological risks. For workers with an exaggerated demonstratively-stuck style of character accentuations from the whole complex of unfavorable environmental factors when working at an oil and gas producing enterprise under shift work organization in the Arctic, the most difficult condition is group isolation. Since it is important for them to pay attention and get support from the inner circle, that is significantly hampered by the involuntary circle of contacts. The need to change the circle of communication, to obtain new information from the external environment under such conditions is not met. At the same time, these workers are well adapted to the conditions of environmental variability, this is an important competence for performing professional tasks in the oil field. Unexpected and emergency situations caused by extreme climatic factors of the environment can occur everyday. Employees of this type are able to cope with them well, quickly, and effectively.

Employees of the second cluster, which have more pronounced anxious-cyclothymical accentuations of character, are characterized by opposite features. They easily cope with the difficulties caused by group isolation. At the same time, effective implementation of professional tasks can be difficult, because they lack the flexibility and quick response to make immediate decisions. However, they can be effective for technical support of the plant and gas pipeline, may calculate the risks of possible malfunctions, develop and carry out maintenance activities, and perform administrative duties for compliance with technology.

We found the absence of statistically significant differences in the level of functional status among representatives from different clusters of character accentuations. This fact can indicate different stylistic features of personnel regulation and requires further study through inclusion in the model of various personality parameters.

The strengths of the study are the definition of two groups of oil and gas workers, depending on the combination of the types of character accentuations. This will allow further study of the features of successful self-regulation and adaptation to the professional activities of workers of both groups. At the same time, this typology needs to be clarified in subsequent scientific research, taking into account the type and characteristics of production, extreme climatic conditions, and professional factors.

Further study of this scientific problem we plan to carry out by definition of the personal resources of employees of both clusters that allow them to reduce psychological risks and effectively perform professional tasks while maintaining physical and mental health.

## 5. Conclusions

1. Workers of the oil and gas producing enterprise, under the shift work organization in Arctic conditions with a moderately pronounced demonstrative type of character accentuation, have favorable functional conditions during the shift period.
2. Workers of oil and gas producing enterprises characterized by different combinations of character accentuations will have different psychological risks, and, consequently, different approaches to their psychological support are needed.
3. On the results of this study, employees of the oil and gas production company under the shift work organization in the Arctic are classified into two clusters, depending on the combination of the expression of types of character accentuations: exalted demonstratively-stuck and anxious-cyclothymic.

## Figures and Tables

**Table 1:** Distribution of the workers by the level of functional status during the shift period.

Group by Functional Status Level	Beginning of the Shift Period (in% of the Surveyed)	Middle of the Shift Period (in% of the Surveyed)	End of the Shift Period (in% of the Surveyed)
Optimal	58.0	66.7	57.1
Unfavorable	42.0	33.3	42.9

**Table 2:** Severity of types of character accentuations of workers.

Types of Character Accentuations	Short Description	Mean	Standard Error of Mean	Standard Deviation
Hyperthymic type	Always elated mood, mobility, activity	16.22	0.544	4.682
Stuck type	Rigidity of affect, “stuck” on the idea	12.38	0.372	3.204
Pedantic type	Rigidity and pedantry	14.54	0.489	4.204
Emotional type	Emotionally labile personality	10.54	0.501	4.307
Anxious type	Anxiety	10.30	0.557	4.791
Cyclothymic type	Periodic change of mood: then apathy, then enthusiasm	14.19	0.389	3.342
Excitable type	Increased impulsive reactivity	11.76	0.479	4.117
Dysthymic type	Low mood background, pessimism	11.84	0.496	4.268
Demonstrative type	Hysterical features	12.76	0.453	3.899
Exalted type	Ability to admire, vivid emotions	14.27	0.854	7.345

Note: 0–6—not expressed; 7–12—moderate; 13–18—the expression is above average; 19–24—accentuated feature [13].

**Table 3:** Results of one-dimensional tests.

Types of Character Accentuations	Sum of Squares of Type III	Degrees of Freedom	Mean Square	F	Significance, *p*
Hyperthymic type	35.962	1	35.962	1.655	0.202
Stuck type	179.604	1	179.604	22.695	<0.001
Pedantic type	44.063	1	44.063	2.546	0.115
Emotional type	132.326	1	132.326	7.796	0.007
Anxious type	450.881	1	450.881	26.510	<0.001
Cyclothymic type	189.509	1	189.509	21.802	<0.001
Excitable type	4.148	1	4.148	0.242	0.624
Dysthymic type	279.159	1	279.159	19.126	<0.001
Demonstrative type	339.680	1	339.680	31.765	<0.001
Exalted type	2201.331	1	2201.331	91.233	<0.001

**Table 4:** Severity of character accentuations in workers of two clusters.

Types of Character Accentuations	Cluster 1		Cluster 2	
	Average Score	Conformity to the Norm	Average Score	Conformity to the Norm
Stuck type	13.89 ± 2.502	Above average	10.78 ± 3.109	Average
Emotional type	11.84 ± 4.978	Average	9.17 ± 2.952	Below average
Anxious type	7.89 ± 4.953	Low	12.83 ± 3.010	Above average
Cyclothymic type	12.63 ± 3.356	Above average	15.83 ± 2.444	Above average
Dysthymic type	9.95 ± 2.799	Below average	13.83 ± 4.663	Above average
Demonstrative type	14.84 ± 2.488	Above average	10.56 ± 3.931	Average
Exalted type	19.58 ± 4.341	High	8.67 ± 5.451	Below average
Name of clusters	Exalted demonstratively-sticking style		Anxious-cyclothymic style	

Note: 0–6—not expressed; 7–12—moderate; 13–18—the expression is above average; 19–24—accentuated feature [13].
